# Does PRGF Work? A Prospective Clinical Study in Dogs with A Novel Polylactic Acid Scaffold Injected with PRGF Using the Modified Maquet Technique

**DOI:** 10.3390/ani11082404

**Published:** 2021-08-14

**Authors:** Victoria Valiño-Cultelli, Óscar Varela-López, Antonio González-Cantalapiedra

**Affiliations:** Department of Anatomy, Animal Production and Veterinary Clinical Sciences, Veterinary Faculty, Universidad de Santiago de Compostela, 27002 Lugo, Spain; oscar.varela@usc.es (Ó.V.-L.); antonio.cantalapiedra@usc.es (A.G.-C.)

**Keywords:** dog, PLA, polylactic acid, TTA, MMT, modified maquet technique, PRGF, PRP, Plasma Rich in Growth Factors, 3D printing

## Abstract

**Simple Summary:**

PRGF is a concentration of autologous platelets in a small volume of plasma, which is performed in a specific way and is an accessible resource in veterinary medicine. The PRGF has multiple demonstrated properties as antimicrobial, analgesic and anti-inflammatory but their osteoinductivity potential is controversial. We decided to use PRGF in combination with a PLA bioresorbable scaffold (a specific type of implant with osteoconduction properties) performed by 3D printing, and personalized for each patient, to determinate if the PRGF can produce osteoinduction and as a result, a faster bone healing and a faster patient recovery. Furthermore, in this study PLA scaffolds are proposed as an alternative for metallic implants to avoid the problems that those can cause. The MMT was the technique selected for solving the RCrCL as it is a variant of TTA that follows the same principle for the correction of the patellar tendon angle to neutralize distractive forces; however, this technique needs a lower amount of metallic implants for the scaffold fixation.

**Abstract:**

Tibial tuberosity advancement is a surgical technique to restore the dynamical stability in the knee by advancing the insertion of the patellar ligament, for which it is necessary to advance the tibial crest, being maintained in the desired position usually by a cage and metallic implants. The purpose of this study was to replace the cage with a polylactic acid biodegradable scaffold designed for each patient by 3D printing, inserting platelet-rich in growth factors (PRGF) to demonstrate its osteoinductive properties. To this end, we used the modified Maquet technique to reduce the amount of metal to a minimum. Fifty-three dogs finished the study. The control and PRGF groups did not present any statistically significant differences in terms of ossification degree (*p* > 0.001) but they demonstrated satisfactory ossification compared to previous publications, although in the PRGF group three of the scaffolds suffered complete reabsorption. The PRGF and control groups did not show any statistically significant differences in terms of lameness degree (*p* > 0.001). However, the PRGF group showed at the first control some analgesic and anti-inflammatory properties but they were not enough for reducing the functional recovery time in a significant way. The PRGF group did not show any complications or negative results associated with their use.

## 1. Introduction

Tibial tuberosity advancement (TTA) is a surgical technique employed to restore the dynamical stability in the knee by neutralizing the tibiofemoral shear forces of the stifle when a patient suffers a rupture of the cranial cruciate ligament (RCrCL) [1,2,3]. It is achieved by advancing the insertion of patellar ligament until it is perpendicular to the tibial plateau with the joint in extension, obtaining a 90° patellar tendon angle (PTA) [2]. For this, the advancement of the tibial crest is necessary, which is usually maintained in this position with a specific plate and cage implants made of stainless steel or titanium [2]. The plate and cage function together as a tension-band construct; the cage prevents the collapse of the osteotomy and loss of desired PTA while the plate neutralizes distractive forces [4]. Once the osteotomy is healed, the implants are not necessary anymore. In veterinary and human medicine, supported by the new technologies such as 3D printing, the new trend is looking for resorbable implants [5,6,7,8,9,10], thus avoiding all the problems that metallic implants may cause [8,11,12]. Moreover, there are several studies on TTA that describe many types of techniques [13], but we decided to use the modified Maquet technique (MMT) for TTA, in an attempt to reduce the amount of metal in the patient.

**Figure 1:**
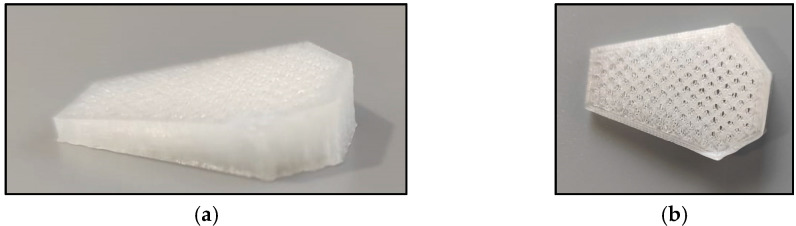
PLA implant. (**a**) Lateral view of the PLA implant. (**b**) Frontal view of the PLA implant.

With that in mind, the aim of this study is to use polylactic acid (PLA) scaffolds carried out by 3D printing to use them as cage implants for TTA (Figure 1). PLA is an aliphatic polyester derived from lactic acid that is thermoplastic and biodegradable, with a great mechanical strength and an excellent biocompatibility [14,15], making it an excellent choice for our purpose.

Platelet-rich plasma (PRP) is a concentration of autologous platelets in a small volume of plasma produced by centrifuging the patient’s blood [16]. Platelets play an important role in primary haemostasis, inducing a complex inflammatory response at the injury site [17], promoting angiogenesis [18] and recruitment of mesenchymal stem cells [19] and are a source of growth and differentiation factors [20]. Those PRP’s growth factors contribute to angiogenesis, macrophage recruitment, chemotaxis of keratinocytes and the mitogenic activity of fibroblasts [21]. Particularly, some of these growth factors are involved in bone regeneration: the fibroblast growth factor induces preosteoblast proliferation and differentiation, the insulin-like growth factor (IGF-1) stimulates the expression of alkaline phosphatase, osteopontin and osteocalcin stimulate bone marrow stromal cells, and platelet-derived growth factor (PDGF) has mitogenic activity and synergy of transforming growth factor (TGF-β1) [22]. The growth factors indirectly activate IL-10, which is an anti-inflammatory cytokine [23]. Furthermore, PRP plays a significant role in angiogenesis (important in bone regeneration), as well as the antimicrobial, analgesic and anti-inflammatory process [21,24,25].

Despite the aforementioned advantages, there is controversial information about the osteoinductive capacity of PRP, since some studies on laboratory animals have not shown any significant influence on this ability [26,27,28], while others have shown a high osteoinductive potential [24,29,30,31]. In addition, if we think in terms of veterinary medicine, the PRP is an accessible and cheap source for our patients that could promote bone healing.

Anitua (2009) has pointed out that the term PRP involved a large number of preparations that differed in the way they were obtained [32]. The Plasma Rich in Growth Factors (PRGF) refers to a product completely autologous and biocompatible, elaborated from the patient’s own blood by only one centrifugation and by using sodic citrate as anticoagulant and calcium chloride as a platelet activator [33], meanwhile for PRP preparations the centrifugation protocols are variated and depend on the author preference. Moreover, the PRP do not require activation with any substance, and thus do not promote the platelet degranulation. Thus, the PRGF is a specific type of PRP that has no white or red blood cells and eliminates proinflammatory activity [34]. To the best of the authors’ knowledge, only one paper in veterinary medicine tested the effect of PRGF in fractures, demonstrating that it helps in bone healing [35].

The decision to use PRGF in this study was based in that we consider that PRGF had a more standardized protocol that allows to compare the properties of this substance with more facility, taking into account that in clinical veterinary medicine there are not many studies that evaluate the clinical properties of this substance in patients, although the studies that exist had promising results [35,36].

Taking all this information into account, our objectives are to first use 3D printed PLA scaffolds for MMT as an alternative to metal implants, in an attempt to avoid the problems that metal may cause. Secondly, to use the PRGF inside of the scaffolds as osteoconductive product and demonstrate its osteoinductive potential on dog patients by accelerating bone healing and patients’ clinical recovery.

## 2. Materials and Methods

### 2.1. Clinical Trial

This prospective study was conducted from December 2017 to July 2020 in the Rof Codina University Veterinary Hospital—Santiago de Compostela University (Lugo, Spain).

The proposed technique with PLA scaffolds was performed in 53 owned dogs. All owners signed a consent form declaring that they were informed about the technique and the new implants, the aleatory use of PRGF, the possibility of complications, the chance of removing the pin and tension band wire, allowing us to use all documentation regarding their dog to be used for scientific research and publication. The owners were aware that they were not going to be informed whether their dog was going to receive PRGF treatment or not.

An anamnesis, physical and traumatological exploration, diagnostic radiographies, complete blood count and biochemistry profiles were determined for each patient. The inclusion and exclusion criteria were previously published [37].

In each case, the surgery was performed by the same expert surgeon and following a standardized protocol. Once the patient fulfils the inclusion criteria, it was randomly assigned to the PRGF or control group and the surgery was scheduled. The scaffold size was obtained by the PTA-TP method [38].

The anaesthetic and analgesic protocol, the antibiotic therapy and the postsurgical therapy were described in a previous paper [37]. The day of the surgery, the premedication for anaesthesia were 10 μg/kg intramuscular (IM) medetomidine and 0.3 mg/kg IM morphine. For the induction, we used 2 mg/kg intravenous (IV) propofol, and during the procedure sevoflurane was employed. Analgesia was obtained by 0.2 mg/kg IV meloxicam and a continuous infusion rate of morphine–lidocaine–ketamine IV. We used cefazolin 22 mg/kg IV as antibiotic, one dose 30 min prior to the surgery and another dose 60 min after the first incision. When the patient was awake, the analgesic continuous infusion rate was continued until discharge, the same day in the afternoon. For post-surgery treatment the protocol was the same for all patients, that is, meloxicam 0.1 mg/kg PO q24 h for 9 days, cefazoline 22 mg/kg PO q8 h for 10 days, digestive protection (depending on patient’s weight) for 10 days. In addition, a Robert Jones bandage was applied from the day of the surgery up to 4 days after, and limited exercise was recommended until follow-up examination.

Once the patient was under premedication effects, if it had been assigned to the PRGF group, the first thing was to place a catheter for a better control of the premedication plan, without introducing fluids or other medication. Secondly, one side of the neck was shaved and aseptically prepared with a surgery field for the extraction of blood. Blood extraction was made with sterile gloves and a 21G butterfly needle with a security extension to a vacutainer citrate sterile tube. We extracted 27 mL from the jugular vein and carried it immediately to the lab for the preparation of PRGF. The patients in the control group did not undergo this step.

After, the hindlimb was aseptically prepared and when the patient was under anaesthesia effects, the standard medial approach for the tibia was used [3] and the technique was performed as described by Valiño-Cultelli (2021) [37]. The scaffolds used were developed in a 3D printer, taking into account patients’ anatomy, as described in the previously mentioned paper.

In case the patient required PRGF, before placing the scaffold, approximately 1.5–2 mL of liquid PRGF was injected with a 2 mL three-body sterile syringe and a 23G needle, uniformly in the scaffold’s holes, and once this was done the implant was placed. The PRGF was activated 5 min before its placement, using a 5% of the volume of CaCl at 10%, hence when the liquid PRGF was placed it had a fluid gel consistency that allowed its placement and maintenance into the scaffold.

**Figure 2:**
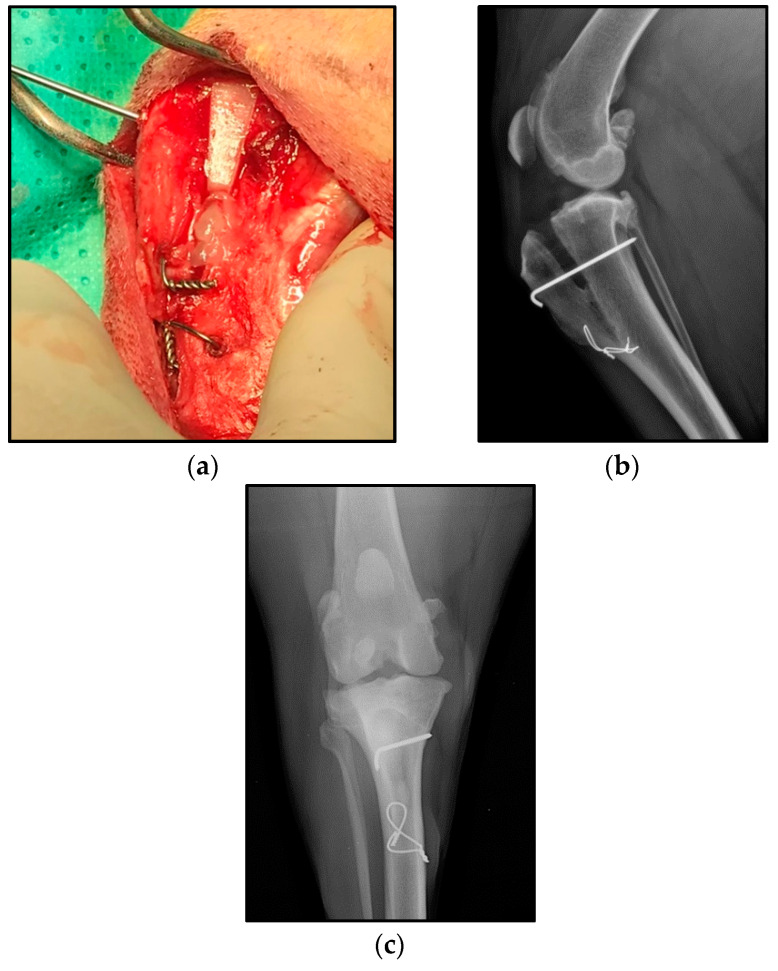
(**a**) PRGF clot placed. (**b**,**c**) Final result of the technique.

After the scaffold was placed and fixed with a metallic pin and a tension band wire [36]; if the patient was in the PRGF group, a PRGF clot was placed in the space left between the scaffold and the distal cortex of the tibial tubercle Figure 2. For the PRGF clot preparation, the PRGF liquid obtained from the patient was activated using a 5% of the total liquid PRGF volume of CaCl at 10%, after it was placed in a heater for 60 min.

The limb was evaluated in order to confirm the absence of positive cranial drawer motion and/or positive cranial tibial thrust and postsurgical radiographs were obtained to assess whether the implants were correctly placed. Finally, the closure of the surgical site was performed as described by Lafaver (2007) [3].

### 2.2. PRGF Fabrication

Blood extraction was made with sterile gloves and a 21G butterfly needle with a security extension to a vacutainer citrate sterile tube. We extracted 27 mL from the jugular vein of the patient and carried it immediately to the lab for the preparation of PRGF.

As described in the technique for the PRGF preparations, the blood in three tubes of 9 mL with citrate was centrifuged at 460 rfc for 8 min at −4 °C [39], using an “Eppendorf 5430 R” centrifuge [39]. The platelet-rich plasma was separated from the clot and the platelet-poor plasma in a laminar flow cabinet, using sterile pipettes and material, avoiding the buffy coat carefully. We obtained approximately 40 μL of PRGF for each 100 μL of total plasma.

Once we have the liquid PRP, the total volume was divided in two equal parts and introduced in two different plain tubes, the quantity obtained from each patient was recorded; the procedure was always performed by the same person. The total quantity of PRGF obtained from each patient was variable, so for the procedure standardization we decided to use 1.5–2 mL for the liquid PRGF that was placed in the scaffold and 1.5–2 mL for the clot preparation that was placed in the space left between the scaffold and the distal cortex of the tibial tubercle.

As described by Anitua (2009) [32], the PRGF was activated before its administration. For the activation of the platelets, we calculated the amount of the 5% of the total volume of PRGF in the plain tube and administrated that 5% of CaCl at 10%, this part of the process inducing the platelet degranulation; the liquid CaCl at 10% was injected in each tube with a syringe and a blue needle in an aseptic way. Out of the two tubes obtained, one was destined for liquid PRGF that was activated in the moment of the surgery, 5 min before its placement, for obtaining a fluid gel that allowed the maintenance of the PRGF in the scaffold; the other one was used to obtain a clot of PRGF, placed in the space left between the scaffold and the distal cortex of the tibial tubercle, by activating the product with CaCl at 10% 60 min before its use and placing it in a heater at 37 °C.

The concentration of platelets was determined in each patient, showing an increase in the number of platelets between blood and the PRGF. Platelet values in PRGF were 1.5–2 times higher than the concentration in blood; the leukocytes present in PRGF presented significant differences between blood and in contrast to plasma-poor in growth factors, it was less than 0.2 × 10^6^/mL in each patient, agreeing with what was previous published by Anitua (2005) [39].

### 2.3. Data Collection

The follow-up was carried out by complete physical examination and radiographs at 1, 2, and 5 months after the surgery. All the radiographs were obtained in laterolateral and caudocranial views and using the same X-ray equipment. In addition, all the observations described by the owner were also recorded.

The data collected were gender, age, body weight, breed, lifestyle, level of exercise difficulty presented by the patient, dates of follow-up radiographs, TTA scaffold size, PRGF application or not, and complications. The data of the PRGF administration were blinded for the follow-up evaluators.

When complications occurred, they were assessed as major or minor complication, according to the previous publications [40]. Those owing to the pin and tension band wire removal, which were previously planned in case the patient needed them, were not taken into consideration.

### 2.4. Radiographic Assessment

Only lateral radiographic projections at 135° were considered when assessing the osteotomy site healing. All patients were randomly assigned a number and their radiographs had the same number; this evaluation was independently performed by two observers that did not know whether the dog had received PRGF or not, using the commercial software OsiriX MD 11.0 (PIXMEO SARL, Geneva, Switzerland) (open-source software; www.osirixviewer.com (accessed on 24 May 2021)).

The evaluation of the osteotomy site healing was conducted using a score on a scale ranging from 0–4, according to a previously published study [41], which we adapted for our technique. The sites were defined as the region of osteotomy proximal to the cage, the region of the cage, and the region of osteotomy distal to the cage. A 0–4 scale was used, with 0 indicating no osseous healing; 1 representing early bone production without bridging between the tibial tuberosity and the shaft of the tibia; 2 indicating bridging bone formation at one site; 3 indicating bridging bone at two sites; 4 representing bridging bone at all three sites.

Follow-up complications and observations were also described.

### 2.5. Lameness Assessment

A numerical rating scale, previously published by Etchepareborde (2011), was used for assessing the lameness in each of the patients [42]. This scale had six levels of lameness severity: 0 = no detectable lameness at a walk or trot and no detectable lateral weight shift at a stance; 1 = no detectable lameness at a walk or trot, and minor lateral weight shift at a stance; 2 = lameness at a walk or trot without hip hike; 3 = lameness at a walk or trot with hip hike; 4 = non-weight-bearing at a trot; 5 = non-weight-bearing at a stance. The degree of lameness was evaluated in the presurgical (PS) stage, at the first follow-up (1fup), second follow-up (2fup) and third follow-up (3fup).

The lameness degree evaluation was independently performed by two observers that did not know whether the patient had received PRGF or not.

### 2.6. Statistical Method

The results were expressed as a mean ± standard deviation, and the statistical analysis was carried out with Sigma Plot 12.5 (Systat software Inc., San Jose, CA, USA). The mean and standard derivation were obtained by descriptive statistics option in all cases. The follow-up days, age, weight, and the used scaffold size were independently compared by *t*-test (*p* < 0.05).

The ossification degrees obtained by the PRGF group and control group at the different follow-ups were compared by ANOVA test (*p* < 0.001).

The lameness degrees obtained by the PRGF group and control group at the different follow-ups were also compared by ANOVA test (*p* < 0.001).

## 3. Results

This study enrolled 53 skeletally mature patients who were operated with MMT and the PLA scaffolds. 24 patients of them were in the control group, without PRGF (18 finished the study) and 29 patients made up the PRGF group (17 finished the study). All the patients that did not finish the study were monitored by phone call.

Among the 53 dogs that completed it, there were 15 cross bread, 6 American Staffordshire Terriers, 6 Golden retrievers, 4 Boxers, 4 Spanish water dogs, 3 Rottweilers, 2 French Bulldogs, 2 Labrador retrievers, 1 Dogo Argentino, 1 Pitbull, 1 American Bulldog, 1 Beagle, 1 Spanish Mastiff, 1 German Shepherd, 1 Cocker Spaniel, 1 Espagneul Breton, 1 Bordeaux Bulldog, 1 Giant Schnauzer, and 1 Galician Paxeiro. There were 33 females, out of whom eight were neutered and 20 males, out of whom four were neutered.

The mean age at the time of the surgery for the 53 patients was 72.86 ± 40.54 months, for the PRGF group (29) 75.10 ± 45.63 months (ranging between 18 and 184 months) and for the control group (24) 70.16 ± 34.18 months (ranging between 21 and 132 months); there were no statistically significant differences in terms of age between the groups (*p* > 0.05).

The mean body weight for the 53 patients was 28.02 ± 10.85 kg, for the PRGF group (29) 26.27 ± 7.41 kg (ranging between 10 and 40 kg) and for the control group (24) 30.13 ± 13.82 kg (ranging between 8.4 and 69.7 kg), there were no statistically significant differences in terms of weight (*p* > 0.05). The mean size of the scaffolds for the 53 patients was 9.30 ± 1.71 mm, for the PRGF group was 9.20 ± 1.52 mm (ranging between 6 and 12 mm) and for the control group was 9.41 ± 1.95 mm (ranging between 5 and 13 mm), there were no statistically significant differences (*p* > 0.05). In 54.71% (29) of the cases, the surgery was performed on the left knee and in 45.28% (24) on the right knee (Table 1).

The mean time of the radiographic follow-ups for the 35 patients that finished the study were 33.74 ± 6.63 days for the first follow-up (1fup), 70.51 ± 11.39 days for the second follow-up (2fup) and 158.68 ± 19.52 for the third follow-up (3fup). For the 1fup, the mean time for the PRGF group was 32.17 ± 5.34 days and for the control group was 35.22 ± 7.51 days; there were no statistically significant differences between groups for the first follow-up (*p* > 0.05). For the 2fup, the mean time for the PRGF group was 68.64 ± 12.98 days and for the control group was 72.27 ± 9.71 days; there were no statistically significant differences between groups for the 2fup (*p* > 0.05). Finally, for the last follow-up, the mean time for the PRGF group was 165.11 ± 14.90 days, whereas for the control group was 152.61 ± 21.74 days; there were no statistically significant differences between groups in terms of the days for the 3fup (*p* > 0.05) (Table 2).

The mean values for the ossification degree of the PRGF group at different times of follow-ups were 1.06 ± 0.96 at 1fup, 2.23 ± 1.14 at 2fup, and 3.29 ± 0.58 at 3fup; there were statistically significant differences in the ossification degrees results between 1fup and 2fup, 1fup and 3 fup, 2fup and 3fup (*p* < 0.05). The mean values for the ossification degree of the control group at different follow-ups were 1.33 ± 1.13 at 1fup, 2.61 ± 0.84 at 2fup, and 3.55 ± 0.51 at 3fup; there were statistically significant differences in the ossification degrees values between 1fup and 2fup, 1fup and 3fup, 2fup and 3fup (*p* < 0.001). When the PRGF and control groups were statistically compared at the same time of follow-up, the results did not present any statistically significant differences neither at 1fup, 2fup nor 3fup (*p* > 0.001) (Table 3). In addition, three patients in the PRGF group presented complete scaffold biodegradation.

The lameness degree mean values were for the PRGF group 3.47 ± 0.94 at PS; 1.29 ± 1.04 at 1fup; 0.64 ± 0.99 at 2fup, and 0.23 ± 0.56 at 3fup; these values presented statistically significant differences between: PS vs. 1fup; PS vs. 2fup; PS vs. 3fup; and 2fup vs. 3fup. In the control group, the lameness degree mean values were 3.55 ± 0.92 at PS; at 1fup the mean value was 2.05 ± 1.39; at 2fup the mean value was 0.83 ± 0.98; and at 3fup was 0.11 ± 0.32, presenting statistically significant differences between follow-ups: PS vs. 1fup; PS vs. 2fup; PS vs. 3fup; 1fup vs. 2fup; and 1fup vs. 3fup (*p* < 0.001). The comparison between the PRGF and control groups showed no statistically significant differences in terms of lameness degree values at the same follow-up time (*p* > 0.001) (Table 4).

Complications were observed in 18.8% (10) of the total number of patients (53). These complications were classified as 5 as major and 5 as minor, according to a previous publication by Cook 2010 [40], complications are described in Table 5. In 4 patients the pin was removed, although this was not considered as a complication because the owners were notified about that possibility before the surgery.

## 4. Discussion

The present study was performed with 53 owned dogs, out of whom 29 were in the PRGF group (with PRGF implemented in the scaffold) and 24 made up the control group (without PRGF). There were 35 patients who finished the study, out of whom 17 were from the PRGF group and 18 were from the control group; the patients who did not complete the follow-ups were monitored by phone call.

The inclusion criteria did not exclude patients due to age, weight, cage size or time between radiographic follow-ups, and there were no statistically significant differences between the PRGF and control group in those parameters, which is something that may affect the results, particularly the ossification [8,41,43,44].

The results in our study demonstrated that PLA scaffolds are an acceptable alternative to metallic implants, agreeing with our preliminary study [37]. In addition, the results in our control group were better than the results obtained in other studies with the same scale in biodegradable implants [4,44]; moreover, our ossification results were also better than another published study with metallic implants [41]. The results in this study were lower than those of another one with metallic implants but in which bone grafts [45] were implemented, taking into account that we only used PRGF that did not show any statistically significant differences with the control group.

As we described in our preliminary study, the maintenance of the advancement with biodegradable implants is controversial [37]. In our study, all the patients maintain the advancement, including the patients with a complete implant biodegradation.

According to our results, we accept our first hypothesis and assume that PLA scaffolds have acceptable conditions to be used in the TTA technique (MMT) instead of the metallic implants, presenting acceptable mechanical conditions and good osteoconductive and osteointegration properties, better than other metallic or biodegradable implants.

**Figure 3:**
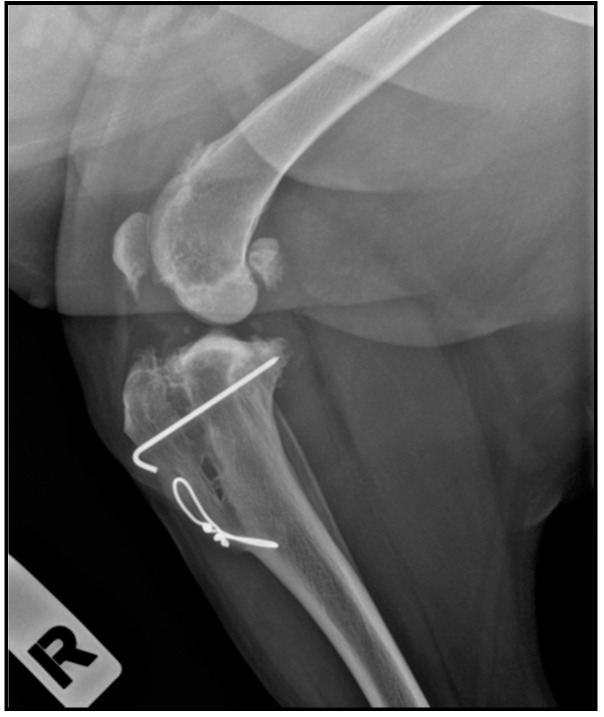
One of the patients that showed complete scaffold reabsorption.

On the other hand, our ossification results indicated no statistically significant differences between the PRGF and the control group, indicating that PRGF had no osteoinductive properties. However, in the control group, none of the scaffolds were fully biodegraded, while in the PRGF group 17.6% of the scaffolds had radiological hints of almost complete biodegradation. One of the patients reached the complete degradation in the second follow-up (2 months after the procedure) and the other two patients at the last follow-up, at 5 months. However, in these three cases, the patients suffered a fracture of the distal cortex of the tibia without displacement, so there is a possibility that the faster ossification was due to the increased tension, which stimulated the mineralization and bone production [44]. But it should be noted that the fractures in the three patients were different, in two of them the fracture of the cortex was iatrogenic during the surgery, in the other case, the patient did not complete the rest period adequately and produced the fracture; thus, the relationship between the cortex fracture and the faster bone healing is not clear. These three cases of total implant biodegradation are the only ones described in a clinical veterinary study on TTA to the best of the authors’ knowledge (Figure 3).

In the control group, there was one patient with the same type of fracture that did not present complete radiological evidence of implant reabsorption, thus we can assume that the PRGF promotes the biodegradation of the PLA scaffold, but it does not influence a faster stabilisation of the osteotomy site (which means a faster clinical recovery). In any case, further studies, with a larger number of patients, are needed.

The PRP has a lot of properties that allow it to be a great osteoinductive product, despite the fact there is controversial information about this [21,24,26,27,28,29,30,31,46].

These controversial results on PRP are justified by the different platelet concentration due to the variety of the PRP protocols and by the type of bone defect, application and/or the animal species [24,47,48,49]; also, the type of biomaterial combined with PRP can influence its efficacy for bone regeneration [48,50]. Except for the first reason, the other ones could justify the reason why as in our study the PRGF did not show any significant results in bone healing, in contrast to the results obtained by López (2019) who demonstrated increased bone healing with the use of the PRGF [35].

Regarding lameness assessment, both the PRGF and control group improved the lameness from presurgical to the last follow-up (Table 4), without statistically significant differences between groups for the same follow-up. 97.1% of the dogs had no detectable lameness at the last follow-up and 85.7% were normal weight bearing at stance. These results are consistent with those of other similar studies that observed no presence of lameness at 12 weeks for 95.38–98.46% of the patients [51,52]; the results are better than those obtained in a previous paper that mentioned 68% resolution of lameness at 12 weeks [53]. Regarding weight bearing, another study observed that at 12 weeks, 80% of the patients had normal weight bearing standing [52], less than in our study; two previous studies noted that TTA improved weight bearing but not always restored it completely [54,55], thus our results are within expectations.

We expected to see an improvement on functional results in the PRGF group because of the PRP anti-inflammatory and analgesic properties in soft tissues [21,35]. If we add to that our hypothesis of a faster bone healing, theoretically it could contribute to decreasing the progression of the muscle atrophy that usually occurs after a TTA intervention [56,57]. However, this atrophy is due to the exercise restrictions that are needed for the stabilisation of the osteotomy before the patient can return to normal activity [58]. Moreover, platelets are viable for only seven days [59], time that is not enough for a long recovery as occurs in patients undergoing a TTA. Nevertheless, we need to consider that in the PRGF group, the mean difference between PS and 1fup is much more than that in the control group (Table 4), due to the PRGF properties.

In addition, it was described that PRP, through the activation of angiogenesis, may cause oedema, discomfort, swelling and inflammation [35,60], but none of these symptoms was observed in our patients on an atypical way by the clinicians in the orthopaedic exploration compared to the control group.

As stated before, the way in which PRP develops its functions depends on many variables and that is important to consider in bone healing, inflammation and the analgesic process. Thus, we reject our second hypothesis because PRGF has not proved its osteoinductive potential, neither does it reduce functional recovery times, though to support this statement, our aim is to continue the research with a larger number of patients and complete the information in this study with histological and CT evidence.

It is important to take into account that one of the limitations of this study was the use of meloxicam intraoperative and in the postsurgery treatment, the decision for this protocol was ethical, to avoid patients’ pain after surgery and ensure patients’ comfort, but this decision could interfere with the PRGF analgesic and anti-inflammatory response during the treatment with NSAIDs for the first 10 days; in any case, our aim was to evaluate the response to PRGF over a long period of time.

Complications were observed in 10 out of 53 patients (18.8%) (Table 5). According to the classification proposed by Cook (2010), five patients presented minor complications and five presented major complications (Table 5). This percentage is within the range of values presented in other papers that studied metallic TTA (11–31.5%) [3,43,61,62].

The tension band wire and pin removal were not considered as complications because they were contemplated in the informed consent as a possibility; four patients had the pin removed, in all cases at the request of the owners.

Within the minor complications, there were fractures of the distal cortex of the tibial crest: two intraoperative and the other two detected at the first follow-up; in the two last cases, the patients did not present lameness and it was a radiological finding; this is one of the most common complications in TTA and MMT. The fractures had no displacement, thus they were solved by strict rest of the patient for a month, as recommended in several studies [3,52,62,63]; in all cases, the evolution was good and they did not require further treatment. Another minor complication was the appearance of vesicles in the area of the incision at the last follow-up, already described in our preliminary study [37]. The treatment was the application of local medication and the administration of antibiotics; the vesicles disappeared with the treatment.

The most common major complication was the tension band wire rupture with or without displacement; in all cases, the owners admitted that they did not follow the movement restriction. In any case, this is one of the most common complications of MMT [64,65]. The less severe case did not present tibial crest displacement, so the patient underwent surgery to replace the tension band wire. In two of the patients, the tension band wire broke and the pin folded, which was detected at the first follow-up. The implant did not have sufficient support and the tibial crest suffered a displacement; in the two cases, the patients underwent another surgery to replace the tension band wire, and the evolution was good. Finally, the last case presented a complicated tibial crest fracture that was solved with surgery by fixing the tibial crest with three pins, the evolution was good in this case too, and the patient did not need any other intervention.

Some authors recommended the replacement of the tension band wire with a metallic conventional plate [42] or by placing another tension band wire caudal to the first one if necessary [66].

**Figure 4:**
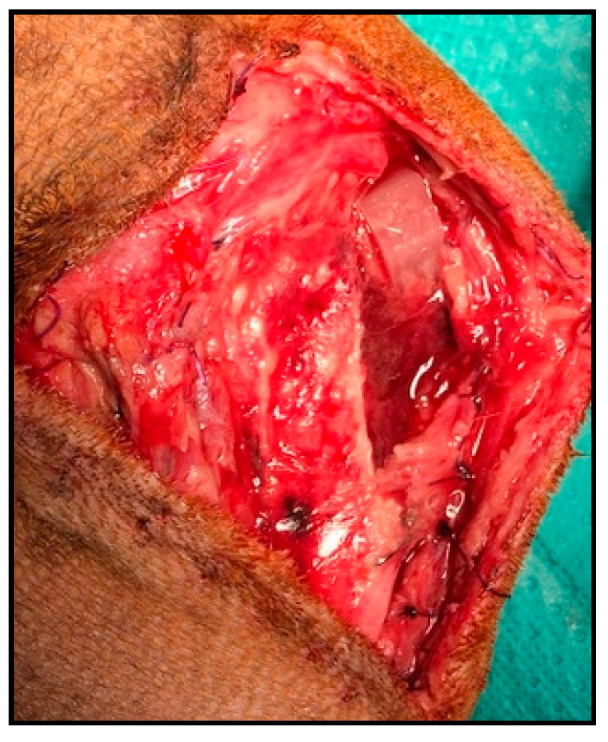
Implant rupture.

Within major complications, one of the patients had an implant rupture, between the third and fourth month after surgery (Figure 4). The patient started with lameness that did not respond to meloxicam and then a fistulous path appeared at the incision site. The decision was to explore the knee by surgery where we saw that the implant was broken, though the fixation had been completed and the advancement was maintained; therefore, we extracted the remaining fragments and made a lavage, and the evolution was good. This is a complication already described in MMT with metallic implants in a previous study, which in that case was solved with rest and NSAID [52].

It is important to mention that some studies noted that the degradation of PLA produced lactic acid, which caused acidification of the extracellular environment with a consequent inflammation that affected the cellular compartment and may lead to the formation of osteolytic zones [9,67]; although other authors indicated that these releases of ions contribute to the proliferation and differentiation of cells into osteogenic phenotypes, which contribute to bone formation [68,69,70,71]. Despite the fact that our study does not yet have any histological evaluation, no signs of inflammation or osteolytic zones were seen on radiographs or detected by exploration in the 53 patients.

No complications associated with PRGF were detected in this study.

## 5. Conclusions

PLA scaffolds are an acceptable alternative to TTA metallic conventional implants, since they showed to be safe and to have a faster grade of bone healing of the osteotomy gap than other previous publications with metallic implants. The rate of complications presented with the PLA scaffolds is also acceptable.

The PRGF used in this study, in combination with PLA scaffolds in dogs, did not prove to have an osteoinductive potential, despite the fact that the biodegradation of the scaffold was faster in three patients of the PRGF group. Our aim is to continue the research with a larger number of patients and complete the information in this study with histological and CT examinations.

The PRGF showed analgesic and anti-inflammatory properties, but they were not enough to reduce the functional recovery time in a significant way.

In addition, the PRGF did not show any complications or negative results associated with their use.

## Figures and Tables

**Table 1:** Patient data.

Groups (*n*)	Age (months)	Weight (kg)	Cage Size (mm)	Affected Knee (*n*) ^1^	Sex (*n*) ^2^
PRGF group (29)				17 (L) 58.62%	18 (F) (6 *n*)
	75.10 ± 45.63 *	26.27 ± 7.41 *	9.20 ± 1.52 *		
				12 (R) 41.37%	11 (M) (2 *n*)
Control group (24)				12 (L) 50%	15 (F) (2 *n*)
	70.16 ± 34.18 *	30.13 ± 13.82 *	9.41 ± 1.95 *	12 (R) 50%	9 (M) (2 *n*)
Total patients (53)				29 (L) 54.71%	33 (F) (8 *n*)
	72.86 ± 40.54	28.02 ± 10.85	9.30 ± 1.71		
				24 (R) 45.28%	20 (M) (4 *n*)

* There were no statistical differences between groups (*p* > 0.05). ^1^ (L) = left knee (R) = right knee. ^2^ (F) = female (M) = male (*n*) = neutered.

**Table 2:** Days follow-up mean for groups.

Follow-Up	PRPGF Group (*n* = 17)	Control Group (*n* = 18)	Total Patient (*n* = 35)
First follow-up (days)	32.17 ± 5.34	35.22 ± 7.51	33.74 ± 6.63
Second follow-up (days)	68.64 ± 12.98	72.27 ± 9.71	70.51 ± 11.39
Third follow-up (days)	165.11 ± 14.90	152.61 ± 21.74	158.68 ± 19.52

There were no statistically significant differences between PRGF and control group in 1fup, 2fup or 3fup.

**Table 3:** *n* of patients by ossification degree.

Ossification Degree	*n* of Patients					
	PRGF Group (*n* = 17)			Control Group (*n* = 18)		
	1Fup	2Fup	3Fup	1Fup	2Fup	3Fup
0	5	1	0	5	0	0
1	8	3	0	6	2	0
2	2	7	1	3	5	0
3	2	3	10	4	9	8
4	0	3	6	0	2	10
Mean	1.06 ± 0.96	2.23 ± 1.14	3.29 ± 0.58	1.33 ± 1.13	2.61 ± 0.84	3.55 ± 0.51

No statistically significant differences between groups for ossification degrees in the same follow-ups time (*p* > 0.001). Scale for evaluation of the healing of the osteotomy site by Hoffmann (2006) [40].

**Table 4:** *n* of patients by lameness degree.

Lameness Degree	*n* of Patients							
	PRGF Group (*n* = 17)				Control Group (*n* = 18)			
	Ps	1 Fup	2 Fup	3 Fup	Ps	1 Fup	2 Fup	3 Fup
0	0	3	11	14	0	3	9	16
1	0	9	2	2	0	3	4	2
2	2	3	3	1	1	5	4	0
3	8	1	1	0	10	5	1	0
4	4	1	0	0	3	1	0	0
5	3	0	0	0	5	1	0	0
Mean	3.47 ± 0.94	1.29 ± 1.04	0.64 ± 0.99	0.23 ± 0.56	3.55 ± 0.92	2.05 ± 1.39	0.83 ± 0.98	0.11 ± 0.32

No statistically significant differences between groups for lameness degree at PS, 1fup, 2fup, 3fup (*p* < 0.001). Scale for evaluation of the lameness degree by Etchepareborde (2011) [41].

**Table 5:** Complications.

	PRGF		Control Group	
Minor	3	Fracture of the distal cortical of the tibial crest without displacement (3)	2	Fracture of the distal cortical of the tibial crest without displacement (1)
				Apparition of vesicles in the incision region (1)
Major	2	Tension band wiring rupture with or without tibial crest displacement (1)	3	Tension band wiring rupture with or without tibial crest displacement (3)
		Implant rupture (1)		

Complication attending to the classification proposed by Cook (2010) [39].

## Data Availability

Not applicable.
